# Finding motif pairs in the interactions between heterogeneous proteins via bootstrapping and boosting

**DOI:** 10.1186/1471-2105-10-S1-S57

**Published:** 2009-01-30

**Authors:** Jisu Kim, De-Shuang Huang, Kyungsook Han

**Affiliations:** 1 School of Computer Science and Engineering, Inha University, Incheon, South Korea; 2 Hefei Institute of Intelligent Machines, Chinese Academy of Sciences, China

## Abstract

**Background:**

Supervised learning and many stochastic methods for predicting protein-protein interactions require both negative and positive interactions in the training data set. Unlike positive interactions, negative interactions cannot be readily obtained from interaction data, so these must be generated. In protein-protein interactions and other molecular interactions as well, taking all non-positive interactions as negative interactions produces too many negative interactions for the positive interactions. Random selection from non-positive interactions is unsuitable, since the selected data may not reflect the original distribution of data.

**Results:**

We developed a bootstrapping algorithm for generating a negative data set of arbitrary size from protein-protein interaction data. We also developed an efficient boosting algorithm for finding interacting motif pairs in human and virus proteins. The boosting algorithm showed the best performance (84.4% sensitivity and 75.9% specificity) with balanced positive and negative data sets. The boosting algorithm was also used to find potential motif pairs in complexes of human and virus proteins, for which structural data was not used to train the algorithm. Interacting motif pairs common to multiple folds of structural data for the complexes were proven to be statistically significant. The data set for interactions between human and virus proteins was extracted from BOND and is available at . The complexes of human and virus proteins were extracted from PDB and their identifiers are available at .

**Conclusion:**

When the positive and negative training data sets are unbalanced, the result via the prediction model tends to be biased. Bootstrapping is effective for generating a negative data set, for which the size and distribution are easily controlled. Our boosting algorithm could efficiently predict interacting motif pairs from protein interaction and sequence data, which was trained with the balanced data sets generated via the bootstrapping method.

## Background

Linear motifs are known to facilitate many protein-protein interactions [1]. Despite the availability of a large volume of data about protein-protein interactions and their sequences, linear motifs are difficult to discover, due to their short length, which is between three and ten amino acids [2]. Recently, several methods have been developed for discovering linear motifs of protein-protein interactions [1,3], but most methods focus on detecting individual linear motifs rather than interacting motif pairs. Motif pairs are more useful than motifs for filtering many spurious protein interactions in current high-throughput data, and for identifying a functional target.

Supervised learning or stochastic methods are often used to predict linear motifs involved in protein-protein interactions. Both negative and positive interactions are required to train the methods. Unlike positive interaction data, negative samples cannot be readily obtained from protein-protein interaction data. Assuming a negative interaction where there is no explicit evidence of a positive interaction results in a much larger negative data set than a positive data set. Such an unbalance between positive and negative data sets makes a prediction biased [4,5]. Generating a negative data set via random selection often does not reflect the original distribution of data, thus it does not produce a good prediction model.

There are a few methods for generating a negative data set. Jansen et al. [6] generate a data set of negative interactions by assuming that proteins in different subcellular compartments of a cell do not interact. However, different subcellular locations only indicate that the proteins have a lower chance of binding than those in the same location, and some proteins are found in more than one subcellular compartment of a cell [7]. The method developed by Gomez et al. [8] assumes a negative protein interaction, if there is no explicit evidence of an interaction. However, this assumption generates a negative data set that is too large, resulting in low sensitivity in interaction predictions. The method that uses the shortest path [7] has difficulty in obtaining a negative data set of the desired size. The method that uses sequence similarity [9] also has difficulty in controlling the size of the negative data set.

In this study, we developed a bootstrapping algorithm for generating a negative data set of protein-protein interactions, and a new boosting algorithm for finding interacting motif pairs from positive and negative data sets. The remainder of the paper describes the algorithms and their experimental results with various parameter values.

## Results and discussion

We measured the prediction performance of the boosting algorithm in terms of sensitivity, specificity and accuracy.

(1) $$Sensitivity=\frac{TP}{TP+FN}$$

(2) $$Specificity=\frac{TN}{TN+FP}$$

(3) $$Accuracy=\frac{TP+TN}{TP+FP+TN+FN}$$

In the following description, the *sampling size S *is the number of negative samples that were examined to generate a single negative data via bootstrapping. When the number of negative samples with *m*-th feature = 1 is greater than the *acceptance ratio A*, the *m*-th feature of the re-sampled negative data is set to 1. The feature vector and the acceptance ratio are described in detail in the method section.

### Affect of acceptance ratios

From the interactions between human and virus proteins, we generated four different negative data sets, by executing the bootstrapping algorithm with four acceptance ratios (1/10, 1/8, 1/6, 1/4). Then, we used both the negative and positive data sets to test the boosting algorithm via five-fold cross validation. Motif pairs predicted from each fold were combined as follows: M_i _= {motif pairs found in at least *i *folds} where i = {1, 2, ..., 5} [7]. Table 1 shows the number of motif pairs predicted with different acceptance ratios.

**Table 1:** Motif pairs found during five-fold cross validation

	A = 1/10	A = 1/8	A = 1/6	A = 1/4
M_1_	12563	21821	50634	142395
M_2_	3479	4866	12472	38008
M_3_	1047	1181	3498	15220
M_4_	189	344	874	6970
M_5_	28	105	141	2134

M_i _denotes a set of motif pairs found in at least *i *folds during five-fold cross validation.

As the acceptance ratio increases, re-sampled negative data have fewer nonzero features, resulting in more motif pairs. This is because the nonzero features of negative data are used to filter out the features that are also nonzero in positive data.

With the sampling size of 120, most non-interaction data were re-sampled to generate a negative data set. We compared the prediction performance of the algorithm with respect to four different acceptance ratios. As shown in Table 2, prediction of motif pairs with a larger acceptance ratio shows a much better performance than that with a smaller acceptance ratio. As the acceptance ratio increases, negative data have more nonzero features. Hence, data with many zero features are easily classified as negative samples.

**Table 2:** Prediction performance with respect to acceptance ratios of bootstrapping

	A = 1/10	A = 1/8	A = 1/6	A = 1/4
Sensitivity	58.35%	75.88%	82.42%	90.42%
Specificity	78.83%	84.40%	92.29%	96.02%
Accuracy	66.09%	80.14%	87.35%	93.22%

As the acceptance ratio A increases, the prediction performance of motif pairs is improved.

### Affect of proportions of positive and negative data sets

**Figure 1:**
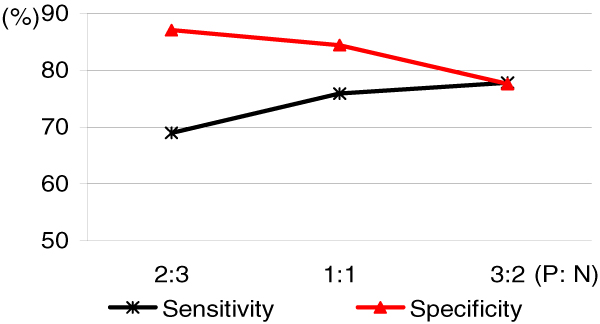
**Sensitivity and specificity of predictions with respect to proportions of positive and the negative data**. As the proportion of positive data increases, the sensitivity increases but the specificity decreases.

For the purpose of comparing the prediction performance with respect to different proportions of positive and negative data sets, we generated three negative data sets with the sampling size of 120 and acceptance ratio of 1/8. The data set for 1,712 interactions between human proteins and virus proteins was used as the positive data set. Table 3 and Figure 1 show the prediction performance with respect to three different proportions of positive and negative data sets. As the proportion of positive data increases, sensitivity increases, but specificity decreases. It is interesting to note that the size of the negative data sets alone affects the performance.

**Table 3:** Prediction performance with respect to proportions of positive and negative data

Data ratio (P: N)	1712: 2283 (2: 3)	1712: 1712 (1: 1)	1712: 1141 (3: 2)
Sensitivity	68.98%	75.88%	77.80%
Specificity	87.03%	84.40%	77.56%
Accuracy	79.30%	80.14%	77.70%

P: positive data, N: negative data.

### Affect of boosting algorithms

The execution time of the boosting algorithm is influenced by the number of hypotheses (T; for Yu's AdaBoost algorithm only), the number of partitioned data sets (S), and the number of randomly selected training data for weak hypotheses (R). Suppose that we set parameters; T = 4, S = 5 and R = 100,000. Yu's AdaBoost uses 5 × 4 = 20 weak hypotheses. But, our boosting algorithm uses only five weak hypotheses. While Yu's AdaBoost uses four weak hypotheses per data set, our boosting algorithm uses only one weak hypothesis per data set. With fewer weak hypotheses than Yu's AdaBoost algorithm, our algorithm has a better performance, as shown in Table 4.

**Table 4:** Prediction performance of two boosting algorithms

Boosting algorithm	AdaBoost algorithm	Our Boosting algorithm
Sensitivity	70.55%	75.88%
Specificity	84.21%	84.40%
Accuracy	77.37%	80.14%

Parameter values: T = 4, S = 5, R = 100,000.

### Motif pairs found in complexes of human and virus proteins

**Figure 2:**
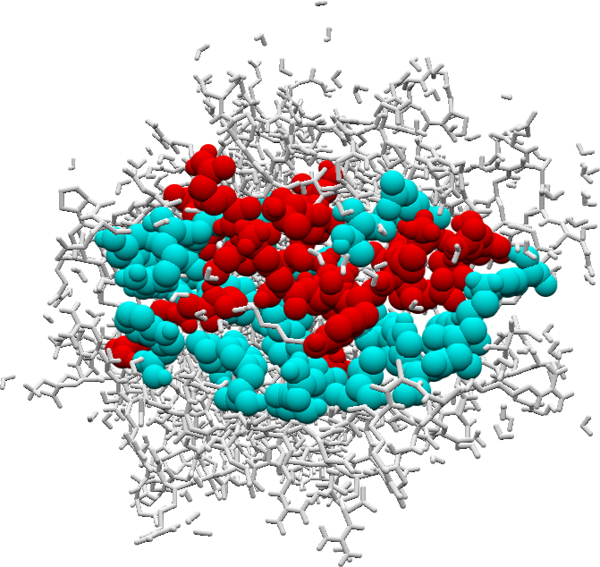
**Motif pairs predicted for 1AGF**. *Red balls*: contact residue pairs correctly predicted, *Cyan balls*: contact residue pairs missed in the prediction, *Gray wireframe*: non-contact residues

Table 5 shows the p-values for each set of motif pairs. The p-value of M_1 _= 1, implying that motif pairs of M_1 _had no more significance than random motif pairs. However, motif pairs of M_2_-M_5 _were more significant than random motif pairs. Figure 2 shows a complex of human and HIV-1 proteins (PDB ID: 1AGF). Among the total of 63 contact residues between chains A and C, 16 residue pairs were included in M_2_.

**Table 5:** Motif pairs found in each fold

Set	# of motif pairs	p-value
M_1_	334	1
M_2_	87	3.13e-3
M_3_	22	3.02e-3
M_4_	7	2.25e-2
M_5_	2	1.79e-1

The number of motif pairs predicted by our boosting algorithm for complexes of human and virus proteins.

## Conclusion

When positive and negative training data sets are unbalanced, the result via the prediction model tends to be biased. We developed a bootstrapping algorithm for generating a negative data set of arbitrary size from protein-protein interaction data. We also developed an efficient boosting algorithm for finding interacting motif pairs in human and virus proteins. The boosting algorithm showed the best performance (84.4% sensitivity and 75.9% specificity) with balanced positive and negative data sets. The boosting algorithm was also used to find potential motif pairs in complexes of human and virus proteins, for which structural data was not used for training the algorithm. Interacting motif pairs common to multiple folds of structural data of complexes were proven to be statistically significant.

This method predicts protein-protein interactions and motif pairs using the protein sequence data. The sequence information alone is insufficient to predict motif pairs for some proteins, but our method provides a useful model for predicting motif pairs in protein-protein interactions when the sequence is the only information available. The data set for interactions between human and virus proteins was extracted from BOND and is available at . The complexes of human and virus proteins were extracted from PDB and their identifiers are available at .

## Methods

### Data set

We extracted the latest data of interactions between human and virus proteins from BOND [10]. As of May, 2008, there were 1,712 interactions between 1,029 human proteins and 603 virus proteins. These interactions were considered as positive data. From 1,712 interactions, we constructed three negative data sets of 2,252, 1,712, and 2,283 samples via the bootstrapping method.

### Feature vector

The way of extracting features in our study was similar to the one used in the studies of Gomez et al. [8] and Yu et al. [7]. In the study by Gomez et al., four-tuple features were used to identify a subsequence of four amino acids. Based on biochemical similarities of amino acids, twenty amino acids were classified into six categories: {IVLM}, {FYW}, {HKR}, {DE}, {QNTP}, and {ACGS} [11]. After classification, there were 6^4 ^= 1,296 possible substrings of length four.

For a given protein sequence, a four-tuple feature is represented as a 1,296-bit binary vector, in which each bit indicates whether the corresponding length-four string occurs in the protein. The encoding scheme for the interaction binary vector is described in Table 6.

**Table 6:** Encoding scheme for the interacting motif pairs

Biochemical property		4-tuple pairs (M bits)		
			Candidate motif pair	
			
Classification	Category number	Bit number	Human 4-tuple	Virus 4-tuple

{I, V, L, M}	0	1	0000	0000
{F, Y, W}	1	2	0000	0001
{H, K, R}	2	⋮	⋮	⋮
{D, E}	3			
{Q, N, T, P}	4	M-1	5555	5554
{A, C, G, S}	5	M	5555	5555

The total number of possible motif pairs is 1,679,616, 1-bit for each motif pair. 1 represents the corresponding motif pair exists in the pair of proteins, and 0 represents the motif pair is absent.

Both our previous study [9] and the study of Yu et al. [7] found interacting motif pairs in yeast proteins. A binary vector representing an interacting motif pair is a palindrome, so the total number M_symmetric _of possible motif pairs is determined by

(4) $$M_{symmetric}=\left(\begin{array}{c} 6^{4}\\ 2 \end{array}\right)+6^{4}=840,456$$

The interactions between human and virus proteins are the interactions between heterogeneous proteins. Hence, the total number M_asymmetric _of possible motif pairs is as follows.

(5) *M*_*asymmetric *_= 6^4^·6^4 ^= 1,679,616

Our method is intended for finding motif pairs with 4 consecutive residues (i, i+1, i+2 and i+3) in each motif. Hence, a motif with non-consecutive residues cannot be found even if the residues are spatially close to each other. Since the total number of possible motif pairs is 6^*m*^·6^*m *^= (6^*m*^)^2 ^= 6^2*m *^for a motif of size *m *(equation 5), the total number of possible motif pairs increases exponentially as the size of *m *increases. The total number of possible motif pairs can be reduced with a motif of a smaller size (e.g., 2 or 3 residues), but the motif of a small size has too many occurrences in the sequences, which significantly reduces the selectivity of the motif.

### Bootstrapping for re-sampling

As in Gomez et al.'s method [8], we assumed a negative interaction if there was no explicit evidence of an interaction. However, this assumption generates a much larger number of negative samples than positive samples. If we randomly select only some of the negative samples, we might miss information from unselected negative samples. Dupret and Koda [5] used bootstrapping to identify the optimal re-sampling proportions in binary classification experiments.

In our study, we used bootstrapping to generate negative data sets via re-sampling negative data. Algorithm 1 describes our bootstrapping method, which is controlled by the sampling size *S *and acceptance ratio *A*. Executing the bootstrapping algorithm yields a single re-sampled negative data from *S *negative data. The re-sampled negative data is represented as a feature vector Y = {y_1_, y_2_, ..., y_M_} via Algorithm 1. The number of 1's in the feature vector Y is controlled by the acceptance ratio *A*. A larger value of *A *produces a feature vector with fewer nonzero elements.

### Algorithm 1 – Bootstrapping algorithm

This algorithm generates the feature vector Y for a single negative data from S samples, where S is the sampling size and A is the acceptance ratio for setting a feature to 1.

1. Randomly sample S protein pairs (P_s1_, P_s2_) with replacement from non-interacting protein pairs, where s = {1, 2, ..., S}.

2. Initialize n_i _= 0 for i = {1, 2, ..., M}

3. Initialize y_i _= 0 for i = {1, 2, ..., M}

4. For s == {1, 2, ..., S}

   a. Make a binary vector X_s _= {x_s1_, x_s2_, ..., x_sM_} for a pair of proteins (P_s1_, P_s2_)

   b. For m = {1...M}

      If x_sm _= 1, n_m _= n_m _+ 1 {n_m _is the number of samples for which the *m*-th feature = 1}

5. For m = {1...M}

      If n_m_/S > A, set y_m _= 1

6. Y = {y_1_, y_2_, ..., y_M_} is a feature vector representing re-sampled negative data.

### The boosting algorithm

In general, the boosting method finds a highly accurate hypothesis by combining weak hypotheses, each of which is only moderately accurate. Typically, each weak hypothesis is a simple classification rule. In AdaBoost (Adaptive Boosting), each weak hypothesis generates not only a classification rule but also a confidence score that estimates the reliability of the classification [12].

The study of Yu et al. [7] uses the AdaBoost algorithm for finding motif pairs in homogeneous protein interactions. One of the differences between Yu's algorithm and ours is the number of weak hypotheses used in the algorithms. In Yu's AdaBoost algorithm, if the weight (α_s1_) of the first weak hypothesis is much greater than the weights of other hypotheses, the final hypothesis is determined mainly by the first weak hypothesis and other hypotheses have negligible effect on the final hypothesis.

Our boosting algorithm determines the weights of weak hypotheses and uses the training data in a different way from Yu's algorithm. While Yu's AdaBoost algorithm uses different weights and the same training data per weak hypothesis, our algorithm uses the same weights and different training data per weak hypothesis. Our boosting algorithm uses fewer weak hypotheses than Yu's algorithm, and requires much less time than their algorithm.

Our algorithm consists of two parts: boosting algorithm and WINNOW2 algorithm. The boosting algorithm described in Algorithm 2 takes as input a training set (x_1_, y_1_), ..., (x_n_, y_n_), where each pair is a binary vector of length M, which represents an interaction with a label in the label set Y. Y = {-1, +1} indicates whether each interaction is positive or negative. The boosting algorithm calls the WINNOW2 algorithm to obtain a weak hypothesis in an iterative series of rounds, where t = {1, ..., S}. In each round, the boosting algorithm computes the weight (α_t_) of the weak hypothesis *h*_*c*,*t*_. The final hypothesis *H*_*t *_for *Set*_*t *_is the weighted sum of weak hypotheses *h*_*c*,*i *_(*i *= 1, ..., *S *and *i *≠ *t*).

We used a regulated stochastic WINNOW2 algorithm [13] with R = 200,000 as a weak classifier (Algorithm 3). The WINNOW2 algorithm is similar to that of Yu et al. [7], except for the step of updating learner factors. Yu's algorithm updates learner factors when x_ki _(feature vector) is 0, but our algorithm updates them when x_ki _is 1. Yu's algorithm takes as input a training set and computes normalized sample weights in each boosting round. In the step of drawing a sample data, data with larger weights are drawn more frequently than those with smaller weights. Since the sample weights are difficult to adjust in each round, our algorithm uses the same weight for every sample and draws samples with equal frequency. But, the training data is changed in every round, and the call to the WINNOW2 algorithm produces different hypotheses according to the training data. Finally, additional regulation is performed to discover effective components. The components with large learner factors are identified as effective components. These effective components are considered as the motif pairs of protein-protein interactions.

**Figure 3:**
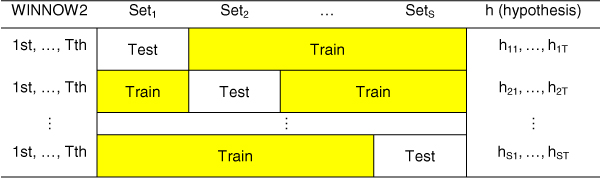
**Framework for Yu's AdaBoost algorithm**. The AdaBoost algorithm requires 20 weak hypotheses for T = 4 and S = 5.

**Figure 4:**
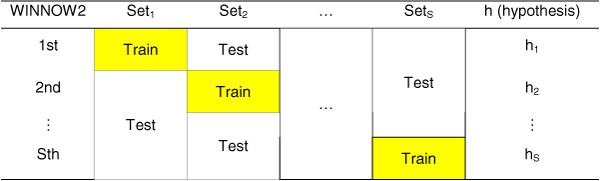
**The framework of our boosting algorithm**. Our algorithm requires only 5 weak hypotheses for S = 5.

Suppose that there are five data sets (S = 5) and four weak hypotheses (T = 4 in Yu's algorithm) per round. Yu's AdaBoost algorithm requires 5 × 4 = 20 weak hypotheses to classify the data. In contrast, our boosting algorithm requires only one weak hypothesis per round, and five weak hypotheses in total, thus it does not need the parameter T. Since the execution times of the algorithms are proportional to the number of hypotheses, our algorithm is more than four times faster than Yu's algorithm for the same data set, without reducing the prediction accuracy [9]. The frameworks for both algorithms are shown in Figures 3 and 4.

### Algorithm 2 – boosting algorithm

The boosting algorithm calls the WINNOW2 algorithm to obtain weak hypotheses. S is the number of divided data sets.

1. Given divided data set *Set*_1_, *Set*_2_, ..., *Set*_*S *_where $\overset{S}{\underset{t=1}{\cup }}Set_{t}=Set_{total}$.

2. For *t *= 1, ..., *S*

   a. Given training data (*x*_1_, *y*_1_), (*x*_2_, *y*_2_), ..., (*x*_*n*_, *y*_*n*_) from *Set*_*t *_where *x*_*i *_∈ {0, 1}^*M*^, *y*_*i *_∈ *Y *= {-1, +1} for {*i *= 1, 2, ..., *n*}

   b. Call the WINNOW2 algorithm to obtain the weak hypothesis *h*_*c*,*t*_.

   c. Compute the error *r*_*t *_of the weak hypothesis *h*_*c*,*t *_at level c.

$$r_{t}=\frac{1}{n}\sum_{i}y_{i}h_{c,t}(x_{i}).$$

   d. Compute the weight *α*_*t *_of the weak hypothesis

$$\alpha_{t}=\frac{1}{2}\ln\left(\frac{1+r_{t}}{1-r_{t}}\right).$$

3. Output the final hypothesis for *Set*_*t*_:

$$H_{t}(x)=sign\sum_{i=1}^{\begin{array}{c} S,i\neq t \end{array}}\alpha_{i}h_{c,i}(x).$$

### Algorithm 3 – WINNOW2 algorithm

The WINNOW2 algorithm trains the weak hypothesis. R is the number of randomly selected data.

1. Given training data (*x*_1_, *y*_1_), (*x*_2_, *y*_2_)..., (*x*_*n*_, y_*n*_).

2. Initialize learner factor *w*_*i *_= 1 for *i *= {1, 2, ..., *M*}, and threshold *θ *= *M*/2

3. For *r *= {1, ..., *R*}

   a. Randomly select a sample data (*x*_*k*_, *y*_*k*_), and let vector *x*_*k *_denote (*x*_*k*1_, *x*_*k*2_, ..., *x*_*kM*_)

   b. The learner responds as follows:

$$\{\begin{array}{lll} h(x_{k})=-1 & if & \sum_{i=1}^{M}w_{i}x_{ki}>\theta \\ h(x_{k})=1 & if & \sum_{i=1}^{M}w_{i}x_{ki}\leq \theta \end{array}$$

   c. Update learner factors $w_{i}=w_{i}2^{x_{ki}(y-h)/2}$

4. Define a regulated classifier *h*_*c *_at level *c *as follows:

$$\{\begin{array}{lll} h_{c}(x_{k})=1 & if & \sum_{i=1}^{M}w_{i,c}x_{ki}>\theta \\ h_{c}(x_{k})=-1 & if & \sum_{i=1}^{M}w_{i,c}x_{ki}\leq \theta \end{array}$$

where *w*_*i*,*c *_= *w*_*i *_if *w*_*i *_≥ *c*, and *w*_*i*,*c *_= 0 otherwise.

5. Let *N*_*c *_denote the number of positive predictions by classifier *h*(*c*) in the training data and *N*_0 _denote the number of positive predictions with the cutoff of 0.

   Output the classifier *h*_*C *_where *C *= arg max {*c *| *N*_*c *_= *N*_0_}.

6. The features with non-zero w_i,c _are effective motif pairs.

## Verification with structural data

To further evaluate the algorithm for the structures of heterogeneous multi-protein complexes, we extracted structural data for complexes of human and virus proteins from PDB [14]. Complexes with RNA or DNA chains were not retrieved. Circa June 2008, there were a total of 105 complexes of human and virus proteins in PDB.

We used five-fold cross validation to evaluate the algorithm. The data set was split into five parts of equal size. The boosting algorithm using the WINNOW2 algorithm for weak hypotheses was trained with one part and tested with the remaining four parts. The train-test procedure consisted of five iterations.

When a residue pair in different chains contained an atomic pair within the distance of 5 Å, we considered the residue pair as a *contact residue pair*. If a motif pair had at least one contact residue pair, we considered the motif pair as a *verifiable motif pair *[7]. To assess the statistical significance of motif pairs predicted by our algorithm, we estimated the p-value of motif pairs by executing Algorithm 4 with *m *= 100,000 [9]. Motif pairs with lower p-values are more significant than those with higher p-values.

### Algorithm 4 – Estimation of p-values of motif pairs

A motif pair with a smaller p-value is more significant than a random motif pair R_i_.

1. Given a set *S *of motif pairs collected by weak hypotheses.

2. Randomly draw *m *motif pairs {*R*_1_, *R*_2_, ..., *R*_*m*_} where *R*_*i *_has the same size as M_k _(k = 1, 2, ...., 5)

3. Compute the p-value of the set S as follows:

$$\begin{array}{cc} p(S)=\frac{\#\left(V\left(R_{i}\right)\geq V\left(S\right)\right)}{m}, & i=\{1,2,...,m\}. \end{array}$$

where *V*(*S*) is the number of verifiable motif pairs.

## Competing interests

The authors declare that they have no competing interests.
